# Active Vaccine Safety Surveillance: Global Trends and Challenges in China

**DOI:** 10.34133/2021/9851067

**Published:** 2021-06-12

**Authors:** Zhike Liu, Ruogu Meng, Yu Yang, Keli Li, Zundong Yin, Jingtian Ren, Chuanyong Shen, Zijian Feng, Siyan Zhan

**Affiliations:** ^1^Department of Epidemiology and Biostatistics, Peking University Health Science Center, Beijing, China; ^2^National Institute of Health Data Science, Peking University, Beijing, China; ^3^National Immunization Programme, Chinese Center for Disease Control and Prevention, Beijing, China; ^4^Center for Drug Reevaluation, National Medical Products Administration, BeijingChina

## Abstract

*Importance*. The great success in vaccine-preventable diseases has been accompanied by vaccine safety concerns. This has caused vaccine hesitancy to be the top 10 in threats to global health. The comprehensive understanding of adverse events following immunization should be entirely based on clinical trials and postapproval surveillance. It has increasingly been recognized worldwide that the active surveillance of vaccine safety should be an essential part of immunization programs due to its complementary advantages to passive surveillance and clinical trials.*Highlights*. In the present study, the framework of vaccine safety surveillance was summarized to illustrate the importance of active surveillance and address vaccine hesitancy or safety concerns. Then, the global progress of active surveillance systems was reviewed, mainly focusing on population-based or hospital-based active surveillance. With these successful paradigms, the practical and reliable ways to create robust and similar systems in China were discussed and presented from the perspective of available databases, methodology challenges, policy supports, and ethical considerations.*Conclusion*. In the inevitable trend of the global vaccine safety ecosystem, the establishment of an active surveillance system for vaccine safety in China is urgent and feasible. This process can be accelerated with the consensus and cooperation of regulatory departments, research institutions, and data owners.

## 1. Introduction

Vaccines are the most cost-effective measures to prevent and control a disease. This can be the essential means for the global elimination or triumph of communicable diseases, such as the nearly eradicated poliovirus, the next priority of cervical cancer [1], or the present threat of the corona virus disease 2019 (COVID-19) pandemic [2]. In addition, personalized vaccinology is emerging, in which newly designed vaccines attempt to overcome more kinds of diseases or health problems, such as aging and cancer [3, 4]. Indeed, preventive or therapeutic vaccines will play a more widespread and critical role in public healthcare in the future.

Unlike other medications, people have much higher expectations for their safety, because vaccines are generally administered to the healthy population. The concerns for vaccine safety are increasingly prominent with the great success in decreasing the epidemic of vaccine-preventable diseases. In 2019, vaccine hesitation, that is, the reluctance or refusal to be vaccinated, despite the availability of vaccines, has been named one of the top 10 threats to global health [5]. This threatens to reverse the progress made for tackling vaccine-preventable diseases or is even associated with the resurgence of eradicated infectious diseases, such as the previous outbreak of measles and pertussis in America [6]. Safety concerns in vaccines are also increasing in China [7]. Hence, these would be the obstacles and challenges to COVID-19 vaccination [8].

Noting vaccine hesitancy is becoming a consistent barrier to vaccination. Comprehensive shreds of evidence from surveillance and empowering individuals with scientific information are meaningful approaches to maintain public confidence and ensure their trust in vaccines [9]. In 2019, the WHO Global Vaccine Safety Blueprint 2.0 (GVSB2.0) prioritized active surveillance and causality assessment for the next 10 years [10] and synchronously developed a global benchmarking tool (GBT) to evaluate national regulatory systems using the maturity level concept, highlighting the assessment of risk-benefit balance and active vigilance activities. The present study aims to review the examples of active surveillance worldwide and its main challenges, and raise more regulatory attention and scientific consensus, which in turn would finally accelerate the robust, responsive, and representative active monitoring in China.

## 2. The Necessity of Active Surveillance

It has been well recognized that studies of phase III clinical trials presently remain as the “gold standard” for the efficacy and safety of vaccines [11]. However, its limits cannot be ignored for vaccine safety, because these commonly recruit well individuals or volunteers as eligible subjects, while barely including special groups with pregnancy or immunocompromised persons. Furthermore, the sample size usually does not reach more than tens of thousands and these are only powered to evaluate commonly known side effects. Moreover, the follow-up time is too short to observe the rare, late-onset, or unexpected adverse events. For example, in Guillain-Barre syndrome, detecting a 2-fold increase in relative risk with a background incidence of 1/100,000 would require a sample of more than 4.7 million subjects, which is impossible in a clinical trial [12]. Furthermore, after a vaccine postlicensure, any important changes, such as the coverage of the target population, should be further evaluated by studies with an independent hypothesis.

In order to minimize risks, the proactive regulation of vaccines was globally implemented in the 1950s and this was latterly evolved into the passive surveillance system by spontaneous reporting adverse events following vaccines. This was also conducted in China after 2005, which consolidated all provinces in Mainland [13]. The prominent advantages are national coverage, open access to the public, and the ability to identify unexpected rare and serious events. However, its weaknesses are also apparent, such as low sensitivity, reporting bias, lack of accurate denominator, and no comparison group to conduct a casual association. For instance, the reporting rate in the Vaccine Adverse Event Reporting System (VAERS) ranged from <1% for rash following the measles, mumps, and rubella vaccine to 3% for hypotonic-hypo-responsive episodes following the whole cell diphtheria-tetanus-pertussis vaccine, reaching up to 68% for polio paralysis following the oral polio vaccine [14].

Given the limitations of these above methods, an increasing number of countries have established or are developing active surveillance using an electronic health database [9]. This refers to the active monitoring of postvaccination clinical manifestations of each administrative dose for all individuals in a defined population, allowing for more precise estimates of the incidence of adverse events and the further determination of the casual relationship. This can be an excellent complement to the passive reporting system [15]. Active surveillance has been a routine and powerful tool that offers scientific and optimal decisions for immunization programs in some developed countries for three decades, such as the Vaccine Safety Datalink (VSD) project in the United States [16] and the Canadian Immunization Monitoring Program, ACTive (IMPACT) [17].

## 3. Framework of the Immunization Safety Surveillance

### 3.1. Update for the Monitoring Framework

**Figure 1:**
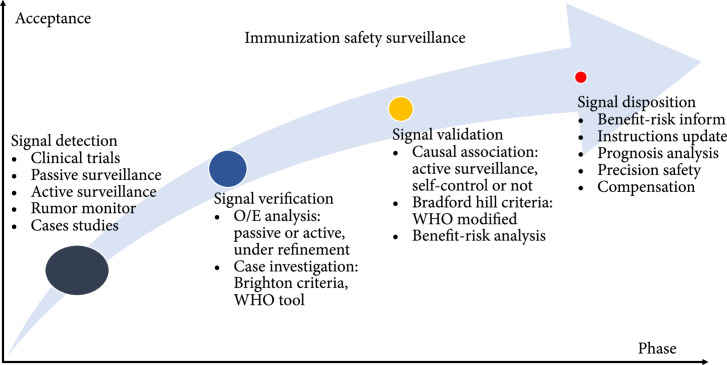
Framework for the immunization safety surveillance: the circle size indicates the number of safety signals or concerns. The more the unresolved vaccine safety concerns, the more likely vaccine hesitation or refusal would occur. The goal is to achieve maximum vaccine safety, in order to reduce phobias and increase acceptance in vaccination. WHO: World Health Organization; O/E: observed over expected.

The vaccine safety signal is defined as information on a newly possible or potential causality relationship between a vaccine and an adverse event or a set of related adverse events determined to have a sufficient likelihood to justify a verificatory action [18]. Previously, postlicensure safety monitoring was classified into three stages: signal detection, signal verification, and signal validation [19]. However, due to the global increase in threats and challenges of vaccine hesitancy and inevitable vaccine inherently-related reactions, the determination of the appropriate and timely response to adverse events has played a critical role in immunization safety surveillance [18]. If a vaccine safety signal is newly determined, quick and effective crisis management should be the priority, in order to lessen its negative impact on healthy individuals and the whole immunization program. Consequently, immunization safety surveillance is supposed to be upgraded to four stages (Figure 1), that is, the addition of signal disposition or crisis management for systematically addressing the potential crisis on safety concern [20]. 

### 3.2. Detection of the Vaccine Safety Signal

#### 3.2.1. Signal Detection or Signal Identification

Vaccine safety signals arise when unanticipated adverse events, which are not predefined in advance, are reported in clinical trials, special studies, social media, or passive surveillance or are identified in active surveillance within a reasonable risk window following immunization. This is a temporal connection between vaccines and adverse events, without confirming the causal relationship [21]. At present, signal detection is mainly performed through passive surveillance worldwide. Hence, it is difficult to timely and accurately detect the safety signal due to the underreported rates and low sensitivity. Thus, improving the convenience of reporting access and stimulating the initiatives of all stakeholders should be the consistent endeavor to enhance the detection ability [10]. Encouragingly, more alternative data sources are being offered to actively monitor the safety signal through the data linkage of vaccination records [22] and these methods provide less-biased information to observe the unusual patterns (e.g., clusters or trends) and the incidence of adverse events. However, this is limited to few developed countries due to the resource-intensive information system, at present.

### 3.3. Verifying the Vaccine Safety Signal

#### 3.3.1. Signal Verification or Signal Refinement

Adverse vaccine reactions may be caused by various reasons, and these were divided into five groups by the Council for International Organizations of Medical Sciences (CIOMS) and WHO: vaccine inherent property-related, vaccine quality defect-related, immunization error-related, immunization anxiety-related, and coincidental event [18]. Therefore, these above safety signals need to be refined to rule out the spurious relations and generate the hypothesis based on biological plausibility, time sequence, and duplicate over time. Observed over expected (O/E) analysis and case investigation are tools to differentiate the temporary findings from potential signals. O/E analysis indicates the comparison of rates of AEFIs observed to what would be expected to alert as a signal, rather than the chance alone [23]. The expected rates of AEFIs can be the background rates of adverse events, similar to those in historical data or the literature [24], or the concurrent rates, similar to those in the parallel control of nonvaccinees or other groups of vaccinees [25]. Mini-Sentinel developed a framework for assessing positive results from the signal refinement in active surveillance, which includes five steps: preparation of a product-specific assessment plan; review of the data validity, descriptive statistics, and program code; conduction of secondary analyses with differing designs; quantitative bias analysis; and interpretation and reporting of the assessment [26]. Performing these in the passive surveillance system remains challenging, because the negative results are subject to bias with underreporting rates and caution should be given to the interpretation [27]. As for case investigations, the Brighton Collaboration developed a set of guidelines for the standardized case definition of AEFIs with certainty levels, especially for serious or rare events [28]. The WHO also developed a standardized algorithm tool for the attribution analysis of AEFIs or named causality assessment at the individual level [29]. These tools would undoubtedly provide guidelines for the definition and classification of AEFIs in a standardized, transparent, and consistent manner to determine the signals, promoting the combined or comparative analysis from different sources.

### 3.4. Validation of the Vaccine Safety Signal

#### 3.4.1. Signal Validation or Signal Confirmation

Once the signal is verified, the hypothesis testing for confirming the causal association between the vaccine and the particular AEFIs should be conducted at the population level, measuring the strength of the association and the consistency with previous studies. Most early researches have performed association analyses using conventional retrospective studies, but this trend has changed with the increase in accessibility of electronic healthcare data in postlicensure surveillance [9]. Four study designs were well recognized and are becoming widespread for vaccine safety surveillance, including self-controlled case series (SCCS), self-controlled risk interval (SCRI), cohorts, and case-control study designs [30]. SCRI and SCCS have been proven to be efficient and rational alternatives to cohorts in a simulation study [31]. Self-controlled designs appear to be more welcome than external-controlled designs due to the advantages in reducing the confounding bias and calculation burden in the big data era. Furthermore, these have been adopted as the primary analysis for vaccine safety assessment in Mini-Sentinel studies [32,33]. In a recent systematic review, the self-controlled design was the most frequently applied to detect the safety signals, followed by the cohort design [22]. Furthermore, in order to control for unmeasured or residual confounding factors, new statistical methods, such as the high-dimensional propensity score, have been proposed in pharmacoepidemiological studies and were more consistent with the results of randomized controlled trials [34]. However, if a safety signal testifies, this should be evaluated in line with the Bradford Hill criteria [18] and a benefit-risk assessment should be further performed before making a reasonable decision.

### 3.5. Disposing the Vaccine Safety Signal

#### 3.5.1. Signal Disposition or Crisis Management

One of the future states of vaccine safety concluded that respondents would like to understand the proactive creation of frameworks, in order to quickly and effectively monitor and respond to crises, focusing on crisis communication plans and risk management [20]. The risks and benefits of vaccines should transparently and proactively inform and communicate with vaccinees or the guardians during any dose vaccination. If a safety signal is newly corroborated, the vaccine instructions should be timely updated and the evidence should be swiftly issued through official news or communication channels, necessarily ahead of misleading media, in order to avoid triggering a crisis [20]. Furthermore, the prognosis analysis on adverse events potentially associated with vaccines is worth exploring. For example, in a prospective cohort study, when comparing vaccine-proximate febrile seizures (VP-FS) with non-vaccine-proximate febrile seizures in children aged below six years, there was no increased risk of prolonged hospitalization, intensive care unit (ICU) admission, seizure duration of more than 15 minutes, or repeat FS within 24 hours [35]. It is convincing that this study can contribute to easing the fear of parents from VP-FS. Suppose that a vaccine with overwhelming benefits, when compared to the risks, and its related adverse event is serious or prone to poor prognosis, studies on precision safety vaccinology, such as genetic predisposition or the prediction model, can individualize the recommendation and minimize the risks [36]. Lastly, regardless of what efforts have been performed to assure the public trust and confidence, considerate welfare must be established for the compensation of vaccines that have caused adverse events, especially for disability or life-threatening diseases, allowing individuals who have accidentally suffered to have supportive development and life quality throughout their lifetime [37].

## 4. Global Progress in Active Safety Surveillance

Given the fundamental functions of active surveillance in the continuous monitoring and quick response to vaccine safety concerns, two pragmatical models have laid the foundation for methodologies in active surveillance systems for vaccine safety. The US Centers for Disease Control and Prevention (CDC) took the lead in conducting VSD in 1898, which was generally named “population-based active surveillance,” because the whole population, whether vaccinated or not, was initially defined in the database, followed by the outcomes linked with vaccination history [16]. Meanwhile, another pioneer, called “hospital-based active surveillance,” was the IMPACT initiated in 1993 in Canada, which daily and actively reviewed all hospital admissions for diseases of interest and related manifestations, confirming the vaccination history [38].

**Figure 2:**
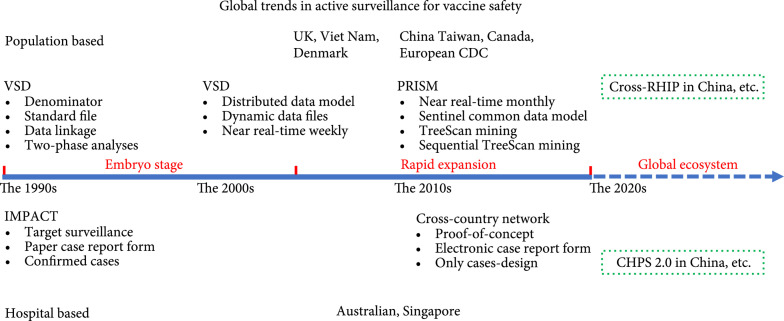
The evolution of active surveillance for global vaccine safety. VSD: the Vaccine Safety Datalink project; PRSIM: Post-Licensure Rapid Immunization Safety Monitoring program; European CDC: European Centre for Disease Prevention and Control; RHIP: Regional Health Information Platform; IMPACT: Immunization Monitoring Program, ACTive; CHPS: China Hospital Pharmacovigilance System.

Without the mutual trust and collaboration from regulatory institutions, academic organizations, and data partners, these systems cannot realize the long-term success. The comparison between these can be observed elsewhere, such as the organization structure, data management, operating mechanism, and primary achievements [9, 39]. The investigators intend to chiefly focus on the emergence, evolution, and lessons of methodologies (Figure 2).

### 4.1. Evolution of Population-Based Active Surveillance

In the 1980s, researchers have begun turning to routine-generated databases that potentially link vaccination with medical outcome records due to the methodological limitations in passive surveillance. In the US, two pilot studies have successfully validated the approach of large-linked databases for vaccine safety study [40], and the record linkage method has been repeatedly demonstrated as an effective approach to immediately identify vaccine-attributable adverse events [41]. It is commendable that the VSD project [40] was well conducted from design to analysis, from the beginning. First, a cohort was integrated from four different health maintenance organization (HMO) databases to evaluate the vaccine safety in children below six years old. Within these HMOs, the source population was similar to the eligible members, allowing the incidence of adverse events after vaccination to be accurately estimated. Second, VSD was initially created as a standardized VSD file applicable to all databases. Furthermore, the 34 outcomes of interest, which consisted of ICD-9 diagnostic codes and relevant information, such as procedures or laboratory tests, were well defined and the list would be amended yearly with new issues. Third, the routine quality checks, the 1–2% periodically testified random sample, and the continuous feedback to each data owner assure the quality of the vaccination and diagnostic data. Lastly, two phases of the analytic strategy were adjusted accordingly for safety detection and safety validation with the available comparison groups. It is well known at present that the project has been proven to be a valuable method for determining crucial evidence on vaccine safety issues, as well as for the cost-effective and optimized immunization policy decisions in the US [42]. 

Since 2000, a series of significant advances have improved the VSD project, which has consolidated its influence in methodologies worldwide [16]. Some innovations are worth highlighting. In order to minimize confidentiality concerns for data sharing, VSD innovatively activated the distributed-data model (DDM), which permits each partner to retain the data on the local server, and the minimum data file for specific studies is securely transferred using encrypted formats. The development of DDM promoted the VSD to transform the data collection method into dynamic data files (DDFs) that can continuously capture the updated data weekly. Finally, the combination of DDM and DDFs empowers the VSD to monitor the vaccine safety near real time, noting that the timeliness of safety research is meaningful for newly licensed vaccines or new vaccine recommendations for existing vaccines. 

Referring to the VSD project, some countries or regions have also started developing active surveillance, such as the UK [43], Denmark [44], Viet Nam [45], Taiwan of China [46], Canada [47], and European CDC [48]. Due to the emergency for influenza vaccine safety surveillance, the Post-Licensure Rapid Immunization Safety Monitoring (PRISM) program was augmented in the US [49], in which 38 million individuals were linked to the national claims database with state or city immunization registries. In order to standardize and structure the partner’s data, PRISM also developed the Sentinel Common Data Model (SCDM 1.0) based on the VSD and others. At present, this was extended to SCDM 7.1.1 for more programs, such as the latest supplement of COVID-19 [50]. PRISM has been endeavoring to develop new statistical methods for the data mining of safety signals. The near real-time surveillance was completed using the monthly updated data [51], and these have exploited multiple TreeScan statistics to explore the potential relationships between a vaccine and a number of unforeseen events at a time [52]. Recently, the combination of these two kinds of methods succeeded and enabled the sequential TreeScan analysis over time [53]. 

In general, population-based active surveillance is information and methodology resource intensive. At present, this has evolved from the primary target on signal validation, into near real-time monitoring and un-predefined safety signal mining. The latter is powerful over timelessness and universality to explore and address vaccine safety concerns, while its accuracy may be disturbed by outcome misclassification or elapsed risk window, lacking symptom surveillance from free text [22]. 

### 4.2. Evolution of Hospital-Based Active Surveillance

In the late 1980s, despite the many advances in improvements, the Canadian passive system continued to fail to detect the increased risk of aseptic meningitis from a new mumps-containing combination vaccine. Hence, it was concluded that the most practical option would be active surveillance for serious vaccination-related adverse events at hospitals [38]. First, the IMPACT planners fully realized that open-ended monitoring for all events following immunization is not feasible due to the lack of systematic information on recent vaccinations. Hence, these targeted the surveillance on priority for all acute neurological admissions and confirmed the completeness of case finding by reviewing the discharge diagnosis codes. Second, effective incentives and continuous encouragement have been given great importance. In order to prevent low-yield searching from discouraging the monitors, a balance between searching for vaccine-related adverse events and finding vaccine-preventable infections was implemented. Intriguingly, the monitoring of vaccine-preventable diseases provided story telling for risk communication on undervaccination or vaccination failures. Strategies, such as permission to take the lead with publications, freedom of additional monitoring targets, and opportunity to be an expertise panel, guarantee team stability and vitality. Third, the standardized case report forms ensure the completeness, consistency, and user friendliness of the project. Finally, IMPACT covered admissions in all areas and contained nearly 90% of the tertiary care pediatric beds. Since 2014, IMPACT started the transition to electronic reporting and managed to integrate the remaining 10% of tertiary care admissions in Canada [17]. 

**Figure 3:**
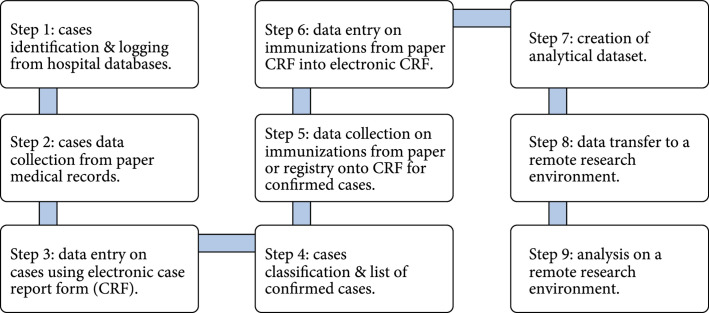
Standardized procedures for the international collaborative hospital-based active surveillance [56].

The modeling on the IMPACT project, Australia [54] and Singapore [55], enhanced the capacity for active surveillance of AEFIs. This appears enormously challenging in low- to middle-income countries to build a robust system for actively monitoring vaccine safety concerns, due to the shortage of the information techniques or enough sample size. However, under the umbrella of the WHO, this dilemma is conquering. Proof-of-concept studies have been repeatedly completed to testify the feasibility and reliability of the international collaborative hospital-based active surveillance on the relationships of rare adverse events and vaccines, using commonly standardized procedures (Figure 3) [56, 57]. These studies were completed with case-only designs, including SCRI, SCCS, and case crossover, due to the lack of population denominators for target vaccines, illustrating the adaptability and efficiency of self-controlled designs. 

## 5. Potentiality of Active Safety Surveillance in China

At present, national passive surveillance is still dominant for vaccine safety surveillance in China. Fortunately, the number of pilot studies and the available databases for actively monitoring vaccine safety are rising. The present study summarized the characteristics of these databases to promote a representatively national active surveillance in China. It has been firmly considered that active surveillance in China would make a difference in global vaccine safety. First, its largest baseline population can timely and convincingly strengthen the vaccine safety efforts around the world. Second, the development of exclusive CDM in Chinese can enhance the innovation of information technology. In addition, the gigantic size and specific characteristics on data may require a novel design or statistical method to address this.

### 5.1. Databases for Hospital-Based Active Surveillance

With the nonessential requirement of linkage with vaccination and successful experiences for other projects, it is feasible to establish a national hospital-based active surveillance for vaccine safety in China. At present, a foundation and essential databases have been established for the rapid application of the IMPACT model. For example, a national hospital-based sentinel network for surveillance of influenza-like illness (HBNS-ILI) has been built across the whole country in 2013 [58], which consisted of outpatient clinics and the emergency outpatients of 554 hospitals and approximately 2.5% of all hospitals in Mainland, China. In line with the IMPACT project, the monitors in HBNS-ILI can simultaneously search for influenza vaccine-associated adverse events, such as GBS, when finding influenza vaccine-preventable illnesses, and subsequently collect the history of influenza vaccines for both of these outcomes. With the combined data of vaccines and adverse events, the safety concerns can be addressed in near real time using these self-controlled designs, or with the combined data of vaccines and ILI, the effectiveness of vaccines can be dynamically monitored using the test-negative study [59] at each influenza-prevailing season. 

Likewise, another victorious exhibition in the China Hospital Pharmacovigilance System (CHPS) [60] is aimed at doing active surveillance for adverse drug reactions (ADR) using real-world electronic medical data in more than 300 hospitals. CHPS adopted the CDM to standardized the data of different partners and utilized the DDM to ensure the security of individual privacy and data files. Furthermore, some novel techniques were developed, such as report assistant, intelligent searching, and ADR sharing to enhance the completeness, timeliness, and accuracy of the ADR identification. In the future, when CHPS extends the CDM to version 2.0 or 3.0, an exclusive module for vaccine-associated adverse events may be imported into this system or a few hospitals in CHPS can start pilot studies for vaccine safety thereafter. Furthermore, the vaccination history may be directly retrieved during the case searching process or the records may be linked with the municipal vaccination registry information system. 

Indeed, previous studies have further illustrated the probability of the above assumptions in China. During the pandemic influenza A (H1N1) in 2009, citywide active surveillance for neurologic diseases following influenza vaccination was implemented in hospitals of Beijing. Within the 10 weeks of mass vaccinations, vaccine recipients observed none of the neurologic conditions, with a total number of 362 incident cases [61]. In addition, a nested case-control study was conducted between 2011 and 2015 in 74 hospitals in Jiangsu. With 1065 incident GBS cases and 4312 parallel controls, there was no increased risk of GBS following the 180 days of influenza vaccine for children and adults [62]. 

### 5.2. Databases for Population-Based Active Surveillance

Since 2012, the statistical information center of the National Health Committee (NHC) has initiated the maturity assessment for Regional Health Information Platforms for the whole population (RHIP) in China [63]. From 2016 to 2019, 38 cities and 43 counties with RHIP have passed through the fourth grade class A assessment, indicating the overall standardized data file, dynamic quality control, and interconnection with most health and medical organizations in the area. The ability of tertiary hospitals in diagnosing and treating rare diseases is more reliable, and these are usually located in the downtown area of a city. Accordingly, the city’s RHIP may be a better choice, when compared to the county’s RHIP, in conducting active surveillance for vaccine safety. By 2019, the RHIP of 38 cities has covered more than 170 million residents and is involved with 14 provinces. 

Recently, a pilot study to evaluate the feasibility of active surveillance of adverse events following human papillomavirus (HPV) vaccination utilized one of the RHIPs in Ningbo [64]. The database covered the entire city and integrated the essential data sources for the active surveillance of the HPV vaccine, including immunization program registration, EMR, resident health records, and death surveillance, and this was inherently linked through a unique national identifier. Within the risk interval of 90 days, the combined incident rate of predefined events was 8.84/100000 doses of bivalent HPV and 3.75/100000 doses of quadrivalent HPV. Thus, the Ningbo RHIP is an available approach to initiate an active surveillance system for adverse events following the HPV vaccine. Furthermore, another investigation study presented that 83.15% of the variables in SCDM 4.0 can be extracted or transformed from the Yinzhou RHIP [65]. 

Indeed, RHIP has the potential for the active surveillance of vaccine safety. However, the establishment of a cross-regional or a national population-based active surveillance system would be confronted by many challenges. The following steps can be the most urgent and notable. Priority should be given in duplicating the feasibility assessment for all the available city RHIPs and knowing all about their data-sharing willingness, informatization progress, coverage completeness, and quality control on the essential data sources mentioned above. Then, some pilot sites should select to form the CDM 1.0 and complete a proof-of-concept study, demonstrating the reliability in hypothesis-confirming studies for several vaccine-event pairs. Lastly, a closely cooperative network must gather data for the creation of the active surveillance system, including the National Medical Products Administration (NMPA), China CDC, universities, and all data partners. Within this network, the NMPA and CDC should be the guider and funder and universities should provide methodology support and innovation. In addition, an efficient mechanism for long-term communication would guarantee its improvement, stability, and sustainability.

## 6. Social and Ethical Considerations

### 6.1. Public Policy for Vaccine Safety

In 2019, “the Vaccine Administration Law of the People’s Republic of China” has been put into effect. The law primarily insists that vaccines are of great importance to public welfare, as well as its life-circle quality management. It requires NHC and NMPA to enhance the AFEI surveillance, especially for those with serious adverse events or enormous social impact, and actualize a dynamic AEFI compensation system. Lately, the “14 ^th^ Five-Year Plan” of China claimed to continue the national expanded immunization program and appealed to construct a global community of health for all, which is in harmony with the WHO’s concept for the global vaccine safety ecosystem [20]. However, both of these policies need to depend on a representative, robust, and sustainable active surveillance system to timely make optimal decisions for any changes. 

### 6.2. Individual Privacy and Research Ethics

With the widespread application of big data, privacy concerns are increasing. The Chinese government has recently issued the “Civil Code” and “Personal Information Protection Law (draft)” to protect individual privacy in the strictest way than ever before. For new laws, personal information, which has been broadly defined as information that can independently or jointly identify natural persons, and anonymization must be unrecognizable and unrestorable standard data. Therefore, it is essential to codevelop methodologies with confidentiality to keep the continuous involvement of the stakeholders. If the data would be transferred from a local site to the centralized server, it must be transformed into a standardized CDM, guaranteeing data security, anonymization, and the minimal risk of reidentification [66]. A statistical method that permits local data storage and the summarization of the distributed analysis results with lossless information can be an alternative [67]. 

In terms of research ethics, independent review and informed consent are the fundamental principles. Some regulations permit broad consent or consent waiver under circumstances with unavailable consent and minimal risks. For these cases, public engagement and deliberative democracy should be taken into consideration [68]. Given that research in a database can only be allowed access to anonymized information, it appears impossible to provide direct benefits to each individual as stakeholders in clinical trials. Hence, the promotion to public health welfare with the funding of studies or blockchains might be able to return the power back to the subjects [69, 70]. 

## 7. Conclusion

Vaccine safety is as significant to human health as vaccine effectiveness. With the inevitable trend of the global vaccine safety ecosystem, some countries or regions have integrated active surveillance as an essential part of the national immunization program. Others have reproduced the success of multiple countries in establishing networks for active surveillance under the support of the WHO. The present study illustrates how to fill the gaps of the active surveillance system for vaccine safety in China, from the perspectives of urgency, necessity, and feasibility.

## Abbreviations

COVID-19: Corona virus disease 2019
GVSB2.0: WHO Global Vaccine Safety Blueprint 2.0
GBT: Global benchmarking tool
AEFI: Adverse events following immunization
VAERS: Vaccine Adverse Event Reporting System
VSD: Vaccine Safety Datalink project
IMPACT: Canadian Immunization Monitoring Program, ACTive
WHO: World Health Organization
CIOMS: Council for International Organizations of Medical Sciences
O/E: Observed over expected
SCCS: Self-controlled case series
SCRI: Self-controlled risk interval
CDC: Centers for Disease Control and Prevention
HMOs: Health maintenance organizations
PRSIM: Post-Licensure Rapid Immunization Safety Monitoring program
CHPS: China Hospital Pharmacovigilance System
RHIP: Regional Health Information Platform
VP-FS: Vaccine-proximate febrile seizures
ICU: Intensive care unit
DDM: Distributed data model
DDF: Dynamic data files
SCDM: Sentinel common data model
ADR: Adverse drug reactions
HBNS-ILI: Hospital-based sentinel network for surveillance of influenza-like illness
NHC: National Health committee
NMPA: National Medical Products Administration
HPV: *Human papillomavirus*.
